# *N*-(2,6-Difluoro­benzo­yl)-*P*,*P*-bis­(pyrrolidin-1-yl)phosphinic amide

**DOI:** 10.1107/S1600536811033216

**Published:** 2011-08-27

**Authors:** Mehrdad Pourayoubi, Atekeh Tarahhomi, Arnold L. Rheingold, James A. Golen

**Affiliations:** a Department of Chemistry, Ferdowsi University of Mashhad, Mashhad, 91779, Iran; b Department of Chemistry, University of California, San Diego, 9500 Gilman, Drive, La Jolla, CA 92093, USA

## Abstract

The phosphoryl and carbonyl groups in the title compound, C_15_H_20_F_2_N_3_O_2_P, are *anti* with respect to each other (but the P- and C-groups are separated by another atom) and the P atom is in a tetra­hedral coordination environment. Two C atoms in one of the pyrrolidinyl fragments are disordered over two sets of sites with occupancies of 0.746 (8) and 0.254 (8). The environments of the pyrrolidinyl N atoms show a slight deviation from planarity and none of the three N atoms is involved in any hydrogen bond as an acceptor. In the crystal, pairs of inter­molecular N—H⋯O hydrogen bonds form inversion dimers.

## Related literature

For hydrogen-bond patterns in compounds containing a C(O)NHP(O) skeleton, see: Toghraee *et al.* (2011 ▶); Pourayoubi *et al.* (2011 ▶). For hydrogen-bond strength, see: Pourayoubi *et al.* (2011 ▶). For a related structure, see: Pourayoubi *et al.* (2010 ▶). For bond lengths, angles and torsion angles, see: Tarahhomi *et al.* (2011 ▶). For graph-set motifs, see Bernstein *et al.* (1995 ▶). For a related phospho­ric triamide, see: Sabbaghi *et al.* (2010 ▶).
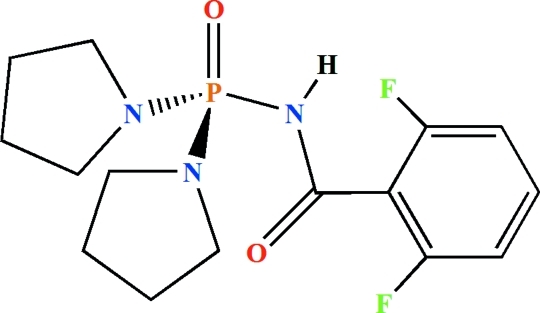


## Experimental

### 

#### Crystal data

C_15_H_20_F_2_N_3_O_2_P
*M* *_r_* = 343.31
Monoclinic, 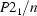

*a* = 10.286 (3) Å
*b* = 14.873 (4) Å
*c* = 10.917 (3) Å
β = 99.296 (3)°
*V* = 1648.3 (7) Å^3^
*Z* = 4
Mo *K*α radiation
μ = 0.20 mm^−1^
*T* = 100 K
0.40 × 0.30 × 0.25 mm

#### Data collection

Bruker APEXII CCD diffractometer
Absorption correction: multi-scan (*SADABS*; Sheldrick, 2004 ▶) *T*_min_ = 0.925, *T*_max_ = 0.952
13279 measured reflections
3776 independent reflections
2953 reflections with *I* > 2σ(*I*)
*R*_int_ = 0.043

#### Refinement

*R*[*F* ^2^ > 2σ(*F* ^2^)] = 0.047
*wR*(*F* ^2^) = 0.119
*S* = 1.05
3776 reflections
230 parameters
7 restraints
H atoms treated by a mixture of independent and constrained refinement
Δρ_max_ = 0.39 e Å^−3^
Δρ_min_ = −0.41 e Å^−3^

### 

Data collection: *APEX2* (Bruker, 2005 ▶); cell refinement: *SAINT* (Bruker, 2005 ▶); data reduction: *SAINT*; program(s) used to solve structure: *SHELXS97* (Sheldrick, 2008 ▶); program(s) used to refine structure: *SHELXL97* (Sheldrick, 2008 ▶); molecular graphics: *SHELXTL* (Sheldrick, 2008 ▶); software used to prepare material for publication: *SHELXTL* and *enCIFer* (Allen *et al.*, 2004 ▶).

## Supplementary Material

Crystal structure: contains datablock(s) I, global. DOI: 10.1107/S1600536811033216/jj2098sup1.cif

Structure factors: contains datablock(s) I. DOI: 10.1107/S1600536811033216/jj2098Isup2.hkl

Additional supplementary materials:  crystallographic information; 3D view; checkCIF report

## Figures and Tables

**Table 1:** Hydrogen-bond geometry (Å, °)

*D*—H⋯*A*	*D*—H	H⋯*A*	*D*⋯*A*	*D*—H⋯*A*
N1—H1*A*⋯O2^i^	0.86 (1)	1.90 (1)	2.757 (2)	175 (2)

Symmetry code: (i) 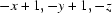.

**Fig. 1.**
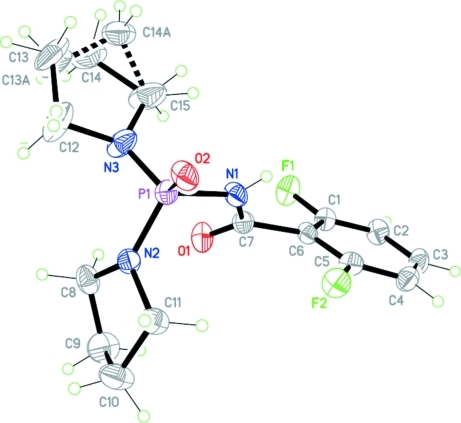
An ORTEP-style plot of title compound with labeling. Displacement ellipsoids are given at 50% probability level and H atoms are drawn as small spheres of arbitrary radii.

## supplementary crystallographic information

### Comment

The patterns of hydrogen bonds and their strengths on phosphoric triamides containing a C(O)NHP(O) skeleton have been discussed (Toghraee *et al.*, 2011; Pourayoubi *et al.*, 2011). The structure determination of the title compound, C_15_H_20_F_2_N_3_O_2_P (Fig. 1), was performed as a continuation of work on this family of compounds in our laboratory.

The carbon atoms C13 and C14 in one of the pyrrolidinyl fragments are disordered over two sets of sites with occupancies of 0.746 (8) and 0.254 (8). The P═O and C═O groups are in *anti* positions with respect to each other. The P atom is in a distorted tetrahedral environment as has been noted for other phosphoric triamides (Sabbaghi *et al.*, 2010). The nitrogen atoms show *sp*^2^ character, the average bond angles at the two tertiary N atoms being 117.8 and 118.7°, respectively.  The P═O, C═O and P—N bond lengths, P—N—C bond angles and O—P—N—C torsion angles are within the expected values (Tarahhomi *et al.*, 2011).

The P═O group and the N—H unit are *syn* with respect to one another.  In the crystal, pairs of intermolecular N—H···O(P) hydrogen bonds (Table 1) form hydrogen-bonded dimers as *R*_2_^2^(8) graph-set rings (Bernstein *et al.*, 1995).

### Experimental

2,6—F_2_C_6_H_3_C(O)NHP(O)Cl_2_ was prepared according to the literature method reported by Pourayoubi *et al.* (2010).

To a solution of 2,6—F_2_C_6_H_3_C(O)NHP(O)Cl_2_ (0.4 g, 1.46 mmol) in dry chloroform (30 ml), a solution of pyrrolidine (0.415 g, 5.84 mmol) in dry chloroform (10 ml) was added at 0 °C. After 4 h stirring, the solvent was removed and the product was washed with distilled water and recrystallized from a mixture of CH_3_OH/DMF (4:1) at room temperature. Single crystals of the title compound were obtained from  this solution  at room temperature.

### Refinement

All non-hydrogen atoms were refined anisotropically by Fourier full matrix least squares on F^2^. Hydrogen atom H1A was located from a Fourier difference map and allowed to refine with a N—H distance of 0.87 (1) Å and *U*_iso_(H) = 1.2*U*_eq_(N). All other hydrogen atoms were placed in geometrically idealized positions with C—H distances of 0.95 Å (aromatic) or 0.99 Å (CH_2_) and with *U*_iso_(H) = 1.2*U*_eq_(C). Carbon atoms C13 and C14 were disordered over two positions with approximate partial occupancies of 0.746 (8) and 0.254 (8). Hydrogen atoms on C12 and C15 were also treated using the above two parts model.

### Crystal data

**Table:**

C_15_H_20_F_2_N_3_O_2_P	*F*(000) = 720
*M**_r_* = 343.31	*D*_x_ = 1.383 Mg m^−^^3^
Monoclinic, *P*2_1_/*n*	Mo *K*α radiation, λ = 0.71073 Å
Hall symbol: -P 2yn	Cell parameters from 7152 reflections
*a* = 10.286 (3) Å	θ = 2.5–27.9°
*b* = 14.873 (4) Å	µ = 0.20 mm^−^^1^
*c* = 10.917 (3) Å	*T* = 100 K
β = 99.296 (3)°	Block, colourless
*V* = 1648.3 (7) Å^3^	0.40 × 0.30 × 0.25 mm
*Z* = 4	

### Data collection

**Table:**

Bruker APEXII CCD diffractometer	3776 independent reflections
Radiation source: fine-focus sealed tube	2953 reflections with *I* > 2σ(*I*)
graphite	*R*_int_ = 0.043
φ and ω scans	θ_max_ = 27.9°, θ_min_ = 2.3°
Absorption correction: multi-scan (*SADABS*; Sheldrick, 2004)	*h* = −13→13
*T*_min_ = 0.925, *T*_max_ = 0.952	*k* = −19→14
13279 measured reflections	*l* = −14→14

### Refinement

**Table:**

Refinement on *F*^2^	Primary atom site location: structure-invariant direct methods
Least-squares matrix: full	Secondary atom site location: difference Fourier map
*R*[*F*^2^ > 2σ(*F*^2^)] = 0.047	Hydrogen site location: inferred from neighbouring sites
*wR*(*F*^2^) = 0.119	H atoms treated by a mixture of independent and constrained refinement
*S* = 1.05	*w* = 1/[σ^2^(*F*_o_^2^) + (0.0419*P*)^2^ + 1.0825*P*] where *P* = (*F*_o_^2^ + 2*F*_c_^2^)/3
3776 reflections	(Δ/σ)_max_ < 0.001
230 parameters	Δρ_max_ = 0.39 e Å^−^^3^
7 restraints	Δρ_min_ = −0.41 e Å^−^^3^

### Special details

**Table:**

Experimental. IR (KBr, ν, cm^-1^): 3062 (NH), 2846, 1684, 1622, 1465, 1442, 1284, 1218, 1181, 1092, 1008, 876, 800, 768, 708, 586.
Geometry. All e.s.d.'s (except the e.s.d. in the dihedral angle between two l.s. planes) are estimated using the full covariance matrix. The cell e.s.d.'s are taken into account individually in the estimation of e.s.d.'s in distances, angles and torsion angles; correlations between e.s.d.'s in cell parameters are only used when they are defined by crystal symmetry. An approximate (isotropic) treatment of cell e.s.d.'s is used for estimating e.s.d.'s involving l.s. planes.
Refinement. Refinement of *F*^2^ against ALL reflections. The weighted *R*-factor *wR* and goodness of fit *S* are based on *F*^2^, conventional *R*-factors *R* are based on *F*, with *F* set to zero for negative *F*^2^. The threshold expression of *F*^2^ > σ(*F*^2^) is used only for calculating *R*-factors(gt) *etc*. and is not relevant to the choice of reflections for refinement. *R*-factors based on *F*^2^ are statistically about twice as large as those based on *F*, and *R*- factors based on ALL data will be even larger.

### Fractional atomic coordinates and isotropic or equivalent isotropic displacement parameters (Å^2^)

**Table:**

	*x*	*y*	*z*	*U*_iso_*/*U*_eq_	Occ. (<1)
P1	0.58132 (4)	0.60634 (4)	0.14444 (5)	0.02623 (15)	
F1	0.06841 (11)	0.66217 (8)	0.14052 (12)	0.0357 (3)	
F2	0.33806 (10)	0.40553 (8)	0.20332 (11)	0.0333 (3)	
O1	0.34013 (13)	0.65942 (10)	0.26101 (14)	0.0340 (4)	
O2	0.65277 (12)	0.54022 (11)	0.07877 (12)	0.0322 (4)	
N1	0.42250 (14)	0.57290 (13)	0.12010 (15)	0.0283 (4)	
H1A	0.402 (2)	0.5349 (12)	0.0604 (16)	0.034*	
N2	0.64224 (15)	0.60885 (11)	0.29142 (15)	0.0260 (4)	
N3	0.57871 (16)	0.71092 (14)	0.10109 (18)	0.0396 (5)	
C1	0.08224 (17)	0.57212 (14)	0.15613 (17)	0.0249 (4)	
C2	−0.02954 (17)	0.52080 (14)	0.15101 (18)	0.0282 (4)	
H2A	−0.1144	0.5478	0.1386	0.034*	
C3	−0.01492 (18)	0.42859 (15)	0.16440 (19)	0.0304 (5)	
H3A	−0.0908	0.3919	0.1621	0.036*	
C4	0.10900 (19)	0.38887 (14)	0.18117 (18)	0.0284 (4)	
H4A	0.1190	0.3256	0.1904	0.034*	
C5	0.21675 (17)	0.44419 (14)	0.18402 (17)	0.0260 (4)	
C6	0.20909 (16)	0.53694 (13)	0.17294 (16)	0.0225 (4)	
C7	0.32904 (17)	0.59659 (14)	0.18895 (17)	0.0249 (4)	
C8	0.6309 (2)	0.68401 (14)	0.3773 (2)	0.0328 (5)	
H8A	0.5548	0.7229	0.3452	0.039*	
H8B	0.7120	0.7209	0.3900	0.039*	
C9	0.6109 (3)	0.63821 (17)	0.4964 (2)	0.0440 (6)	
H9A	0.5167	0.6244	0.4962	0.053*	
H9B	0.6438	0.6761	0.5695	0.053*	
C10	0.6916 (3)	0.55236 (17)	0.4966 (2)	0.0450 (6)	
H10A	0.6603	0.5057	0.5497	0.054*	
H10B	0.7862	0.5641	0.5265	0.054*	
C11	0.6688 (2)	0.52362 (14)	0.36086 (18)	0.0334 (5)	
H11A	0.7477	0.4934	0.3391	0.040*	
H11B	0.5926	0.4823	0.3431	0.040*	
C12	0.7037 (2)	0.75855 (18)	0.0940 (3)	0.0512 (7)	
H12A	0.7311	0.7953	0.1693	0.061*	0.746 (8)
H12B	0.7748	0.7152	0.0857	0.061*	0.746 (8)
H12C	0.7680	0.7205	0.0597	0.061*	0.254 (8)
H12D	0.7447	0.7840	0.1748	0.061*	0.254 (8)
C13	0.6757 (6)	0.8164 (5)	−0.0173 (6)	0.0601 (19)	0.746 (8)
H13A	0.6871	0.7829	−0.0933	0.072*	0.746 (8)
H13B	0.7335	0.8700	−0.0094	0.072*	0.746 (8)
C14	0.5338 (4)	0.8424 (3)	−0.0195 (4)	0.0520 (13)	0.746 (8)
H14A	0.4933	0.8646	−0.1024	0.062*	0.746 (8)
H14B	0.5252	0.8890	0.0434	0.062*	0.746 (8)
C13A	0.6387 (15)	0.8325 (12)	0.0017 (13)	0.043 (4)	0.254 (8)
H13C	0.6062	0.8819	0.0495	0.051*	0.254 (8)
H13D	0.7061	0.8577	−0.0438	0.051*	0.254 (8)
C14A	0.5278 (10)	0.7968 (8)	−0.0887 (9)	0.048 (3)	0.254 (8)
H14C	0.5559	0.7543	−0.1492	0.057*	0.254 (8)
H14D	0.4704	0.8443	−0.1318	0.057*	0.254 (8)
C15	0.4701 (2)	0.7512 (2)	0.0127 (3)	0.0557 (8)	
H15A	0.3910	0.7613	0.0518	0.067*	0.746 (8)
H15B	0.4462	0.7131	−0.0620	0.067*	0.746 (8)
H15C	0.4195	0.7936	0.0565	0.067*	0.254 (8)
H15D	0.4095	0.7034	−0.0250	0.067*	0.254 (8)

### Atomic displacement parameters (Å^2^)

**Table:**

	*U*^11^	*U*^22^	*U*^33^	*U*^12^	*U*^13^	*U*^23^
P1	0.0153 (2)	0.0358 (3)	0.0272 (3)	−0.00273 (19)	0.00216 (18)	0.0048 (2)
F1	0.0267 (6)	0.0268 (7)	0.0548 (8)	0.0024 (5)	0.0109 (5)	−0.0006 (6)
F2	0.0230 (5)	0.0356 (7)	0.0407 (7)	0.0057 (5)	0.0034 (5)	0.0040 (5)
O1	0.0278 (7)	0.0339 (9)	0.0410 (8)	−0.0075 (6)	0.0079 (6)	−0.0103 (7)
O2	0.0182 (6)	0.0502 (10)	0.0279 (7)	−0.0018 (6)	0.0030 (5)	−0.0034 (7)
N1	0.0187 (7)	0.0436 (11)	0.0230 (8)	−0.0054 (7)	0.0046 (6)	−0.0037 (8)
N2	0.0261 (8)	0.0215 (9)	0.0284 (8)	−0.0003 (6)	−0.0019 (6)	0.0007 (7)
N3	0.0218 (8)	0.0465 (12)	0.0508 (12)	0.0006 (8)	0.0067 (7)	0.0229 (10)
C1	0.0244 (8)	0.0237 (10)	0.0277 (9)	0.0011 (7)	0.0079 (7)	−0.0022 (8)
C2	0.0179 (8)	0.0351 (12)	0.0325 (10)	−0.0003 (8)	0.0067 (7)	−0.0042 (9)
C3	0.0251 (9)	0.0355 (12)	0.0318 (10)	−0.0099 (8)	0.0083 (8)	−0.0042 (9)
C4	0.0305 (9)	0.0264 (11)	0.0291 (10)	−0.0035 (8)	0.0074 (8)	0.0001 (8)
C5	0.0215 (8)	0.0323 (11)	0.0242 (9)	0.0032 (8)	0.0035 (7)	0.0001 (8)
C6	0.0180 (8)	0.0289 (11)	0.0213 (9)	−0.0024 (7)	0.0055 (7)	−0.0018 (8)
C7	0.0189 (8)	0.0306 (11)	0.0250 (9)	−0.0026 (7)	0.0027 (7)	0.0014 (8)
C8	0.0306 (10)	0.0233 (11)	0.0408 (12)	0.0019 (8)	−0.0047 (8)	−0.0049 (9)
C9	0.0621 (15)	0.0340 (13)	0.0371 (12)	0.0038 (11)	0.0115 (11)	−0.0074 (10)
C10	0.0674 (16)	0.0360 (14)	0.0287 (11)	0.0082 (12)	−0.0009 (11)	−0.0024 (10)
C11	0.0458 (12)	0.0254 (11)	0.0272 (10)	0.0066 (9)	0.0004 (9)	−0.0005 (9)
C12	0.0343 (11)	0.0389 (15)	0.085 (2)	−0.0063 (10)	0.0237 (12)	0.0122 (14)
C13	0.091 (4)	0.038 (3)	0.064 (3)	−0.025 (3)	0.052 (3)	−0.008 (2)
C14	0.081 (3)	0.040 (2)	0.036 (2)	−0.0016 (19)	0.0119 (19)	0.0143 (18)
C13A	0.048 (8)	0.046 (9)	0.035 (7)	−0.004 (6)	0.010 (6)	0.013 (6)
C14A	0.072 (7)	0.037 (6)	0.032 (6)	0.001 (5)	0.004 (5)	0.007 (5)
C15	0.0462 (13)	0.068 (2)	0.0523 (16)	0.0092 (13)	0.0052 (12)	0.0353 (14)

### Geometric parameters (Å, °)

**Table:**

P1—O2	1.4803 (16)	C9—H9B	0.9900
P1—N3	1.625 (2)	C10—C11	1.524 (3)
P1—N2	1.6261 (17)	C10—H10A	0.9900
P1—N1	1.6872 (16)	C10—H10B	0.9900
F1—C1	1.355 (2)	C11—H11A	0.9900
F2—C5	1.359 (2)	C11—H11B	0.9900
O1—C7	1.215 (2)	C12—C13	1.479 (7)
N1—C7	1.359 (2)	C12—C13A	1.566 (14)
N1—H1A	0.863 (9)	C12—H12A	0.9900
N2—C8	1.476 (3)	C12—H12B	0.9900
N2—C11	1.480 (3)	C12—H12C	0.9891
N3—C15	1.480 (3)	C12—H12D	0.9903
N3—C12	1.481 (3)	C13—C14	1.507 (7)
C1—C2	1.374 (3)	C13—H13A	0.9900
C1—C6	1.390 (2)	C13—H13B	0.9900
C2—C3	1.385 (3)	C14—C15	1.571 (4)
C2—H2A	0.9500	C14—H14A	0.9900
C3—C4	1.390 (3)	C14—H14B	0.9900
C3—H3A	0.9500	C13A—C14A	1.481 (15)
C4—C5	1.377 (3)	C13A—H13C	0.9900
C4—H4A	0.9500	C13A—H13D	0.9900
C5—C6	1.386 (3)	C14A—C15	1.500 (9)
C6—C7	1.507 (2)	C14A—H14C	0.9900
C8—C9	1.512 (3)	C14A—H14D	0.9900
C8—H8A	0.9900	C15—H15A	0.9900
C8—H8B	0.9900	C15—H15B	0.9900
C9—C10	1.523 (3)	C15—H15C	0.9892
C9—H9A	0.9900	C15—H15D	0.9898
			
O2—P1—N3	118.75 (10)	C13—C12—N3	105.4 (3)
O2—P1—N2	110.54 (8)	N3—C12—C13A	94.9 (6)
N3—P1—N2	104.53 (10)	C13—C12—H12A	110.7
O2—P1—N1	105.77 (9)	N3—C12—H12A	110.7
N3—P1—N1	105.44 (9)	C13A—C12—H12A	100.4
N2—P1—N1	111.79 (8)	C13—C12—H12B	110.7
C7—N1—P1	126.16 (14)	N3—C12—H12B	110.7
C7—N1—H1A	118.4 (15)	C13A—C12—H12B	130.1
P1—N1—H1A	115.3 (15)	H12A—C12—H12B	108.8
C8—N2—C11	110.53 (16)	C13—C12—H12C	94.3
C8—N2—P1	125.92 (13)	N3—C12—H12C	112.7
C11—N2—P1	119.72 (13)	C13A—C12—H12C	113.6
C15—N3—C12	110.05 (19)	H12A—C12—H12C	120.9
C15—N3—P1	123.53 (17)	C13—C12—H12D	120.5
C12—N3—P1	119.96 (15)	N3—C12—H12D	112.6
F1—C1—C2	118.29 (16)	C13A—C12—H12D	112.4
F1—C1—C6	117.78 (16)	H12B—C12—H12D	96.8
C2—C1—C6	123.90 (19)	H12C—C12—H12D	110.0
C1—C2—C3	118.04 (17)	C12—C13—C14	102.8 (4)
C1—C2—H2A	121.0	C12—C13—H13A	111.2
C3—C2—H2A	121.0	C14—C13—H13A	111.2
C2—C3—C4	121.09 (18)	C12—C13—H13B	111.2
C2—C3—H3A	119.5	C14—C13—H13B	111.2
C4—C3—H3A	119.5	H13A—C13—H13B	109.1
C5—C4—C3	117.83 (19)	C13—C14—C15	102.3 (4)
C5—C4—H4A	121.1	C13—C14—H14A	111.3
C3—C4—H4A	121.1	C15—C14—H14A	111.3
F2—C5—C4	117.79 (18)	C13—C14—H14B	111.3
F2—C5—C6	118.20 (16)	C15—C14—H14B	111.3
C4—C5—C6	123.97 (17)	H14A—C14—H14B	109.2
C5—C6—C1	115.16 (16)	C14A—C13A—C12	112.3 (12)
C5—C6—C7	122.82 (16)	C14A—C13A—H13C	109.1
C1—C6—C7	121.82 (18)	C12—C13A—H13C	109.1
O1—C7—N1	123.83 (17)	C14A—C13A—H13D	109.1
O1—C7—C6	121.16 (17)	C12—C13A—H13D	109.1
N1—C7—C6	114.99 (17)	H13C—C13A—H13D	107.9
N2—C8—C9	103.94 (17)	C13A—C14A—C15	91.4 (9)
N2—C8—H8A	111.0	C13A—C14A—H14C	113.4
C9—C8—H8A	111.0	C15—C14A—H14C	113.4
N2—C8—H8B	111.0	C13A—C14A—H14D	113.4
C9—C8—H8B	111.0	C15—C14A—H14D	113.4
H8A—C8—H8B	109.0	H14C—C14A—H14D	110.7
C8—C9—C10	103.24 (19)	N3—C15—C14A	108.5 (4)
C8—C9—H9A	111.1	N3—C15—C14	101.4 (2)
C10—C9—H9A	111.1	N3—C15—H15A	111.5
C8—C9—H9B	111.1	C14A—C15—H15A	134.5
C10—C9—H9B	111.1	C14—C15—H15A	111.5
H9A—C9—H9B	109.1	N3—C15—H15B	111.5
C9—C10—C11	103.61 (18)	C14A—C15—H15B	74.1
C9—C10—H10A	111.0	C14—C15—H15B	111.5
C11—C10—H10A	111.0	H15A—C15—H15B	109.3
C9—C10—H10B	111.0	N3—C15—H15C	110.1
C11—C10—H10B	111.0	C14A—C15—H15C	111.7
H10A—C10—H10B	109.0	C14—C15—H15C	80.1
N2—C11—C10	104.16 (17)	H15B—C15—H15C	133.1
N2—C11—H11A	110.9	N3—C15—H15D	109.8
C10—C11—H11A	110.9	C14A—C15—H15D	108.4
N2—C11—H11B	110.9	C14—C15—H15D	141.7
C10—C11—H11B	110.9	H15A—C15—H15D	77.4
H11A—C11—H11B	108.9	H15C—C15—H15D	108.3
			
O2—P1—N1—C7	160.11 (17)	P1—N1—C7—C6	−162.13 (14)
N3—P1—N1—C7	−73.26 (19)	C5—C6—C7—O1	−126.8 (2)
N2—P1—N1—C7	39.7 (2)	C1—C6—C7—O1	47.8 (3)
O2—P1—N2—C8	157.70 (16)	C5—C6—C7—N1	51.5 (2)
N3—P1—N2—C8	28.81 (18)	C1—C6—C7—N1	−133.96 (19)
N1—P1—N2—C8	−84.76 (18)	C11—N2—C8—C9	−16.4 (2)
O2—P1—N2—C11	−46.49 (17)	P1—N2—C8—C9	141.28 (16)
N3—P1—N2—C11	−175.38 (15)	N2—C8—C9—C10	33.4 (2)
N1—P1—N2—C11	71.05 (17)	C8—C9—C10—C11	−38.2 (2)
O2—P1—N3—C15	95.4 (2)	C8—N2—C11—C10	−7.4 (2)
N2—P1—N3—C15	−140.9 (2)	P1—N2—C11—C10	−166.62 (15)
N1—P1—N3—C15	−22.9 (2)	C9—C10—C11—N2	28.0 (2)
O2—P1—N3—C12	−53.6 (2)	C15—N3—C12—C13	−11.0 (4)
N2—P1—N3—C12	70.2 (2)	P1—N3—C12—C13	141.7 (3)
N1—P1—N3—C12	−171.80 (19)	C15—N3—C12—C13A	5.5 (7)
F1—C1—C2—C3	178.66 (18)	P1—N3—C12—C13A	158.3 (6)
C6—C1—C2—C3	0.5 (3)	N3—C12—C13—C14	33.6 (5)
C1—C2—C3—C4	−0.7 (3)	C13A—C12—C13—C14	−25 (2)
C2—C3—C4—C5	0.0 (3)	C12—C13—C14—C15	−42.7 (5)
C3—C4—C5—F2	178.50 (17)	C13—C12—C13A—C14A	89 (3)
C3—C4—C5—C6	0.9 (3)	N3—C12—C13A—C14A	−35.5 (12)
F2—C5—C6—C1	−178.62 (16)	C12—C13A—C14A—C15	47.7 (13)
C4—C5—C6—C1	−1.1 (3)	C12—N3—C15—C14A	23.9 (6)
F2—C5—C6—C7	−3.8 (3)	P1—N3—C15—C14A	−127.7 (5)
C4—C5—C6—C7	173.80 (18)	C12—N3—C15—C14	−15.1 (3)
F1—C1—C6—C5	−177.82 (17)	P1—N3—C15—C14	−166.7 (2)
C2—C1—C6—C5	0.3 (3)	C13A—C14A—C15—N3	−40.9 (10)
F1—C1—C6—C7	7.3 (3)	C13A—C14A—C15—C14	44.1 (8)
C2—C1—C6—C7	−174.62 (18)	C13—C14—C15—N3	35.1 (4)
P1—N1—C7—O1	16.1 (3)	C13—C14—C15—C14A	−70.4 (8)

### Hydrogen-bond geometry (Å, °)

**Table:**

*D*—H···*A*	*D*—H	H···*A*	*D*···*A*	*D*—H···*A*
N1—H1A···O2^i^	0.86 (1)	1.90 (1)	2.757 (2)	175 (2)

Symmetry codes: (i) −*x*+1, −*y*+1, −*z*.
